# Sexual harassment and abuse; disclosure and awareness of report- and support resources in Norwegian sport- and non-sport high schools: a prospective exploratory study

**DOI:** 10.3389/fpsyg.2023.1168423

**Published:** 2023-07-14

**Authors:** Nina Sølvberg, Monica Klungland Torstveit, Margo Mountjoy, Jan H. Rosenvinge, Gunn Pettersen, Jorunn Sundgot-Borgen

**Affiliations:** ^1^Department of Sports Medicine, The Norwegian School of Sport Sciences, Oslo, Norway; ^2^Department of Sport Science and Physical Education, Faculty of Health and Sport Science, University of Agder, Kristiansand, Norway; ^3^Department of Family Medicine, McMaster University, Hamilton, ON, Canada; ^4^International Olympic Committee Working Group on the Prevention of Harassment and Abuse in Sport, Lausanne, Switzerland; ^5^International Research Network on Violence and Integrity in Sport, Antwerp, Belgium; ^6^Department of Psychology, UiT - The Arctic University of Norway, Tromsø, Norway; ^7^Department of Health and Care Sciences, UiT - The Arctic University of Norway, Tromsø, Norway

**Keywords:** sexual harassment (MeSH), sexual abuse, disclosure (mesh), report, support

## Abstract

**Purpose:**

To examine high school students’ disclosure of sexual harassment and abuse (SHA), and awareness of reporting systems and support mechanisms in school among students, leaders, and coaches.

**Method:**

Norwegian 17-year-old high school elite athletes (*n* = 630), recreational athletes (*n* = 307), and reference students (*n* = 263) responded to an online questionnaire at two measurement points, 1 year apart (T1 and T2). Leaders and coaches (*n* = 249) at the participating high schools responded to an adapted version of the questionnaire at T1. Data were analyzed using ANOVA or Welch test, Pearson Chi-Square test, and McNemar test.

**Results:**

In total, 11.4 and 34.0% of the adolescents were aware of reporting systems and support mechanisms, respectively, in their schools. Nearly all the leaders, and half of the coaches were aware of these resources. Among the adolescents with lifetime experience of SHA, 20.1% had disclosed their experiences to someone. Girls disclosed more frequently than boys. The elite- and recreational athletes disclosed less often compared with the reference students. A negative change from T1 to T2 was found in disclosure of SHA and awareness of support mechanisms. At T2, 6.5% of the adolescents reported that their school had implemented measures against SHA during the last 12 months.

**Conclusion:**

The results emphasize a need for institutional effort to improve information about available report- and support resources and increase the relevance of use of such systems for adolescents.

## Introduction

Sexual harassment and abuse (SHA) are societal problems affecting all genders and age groups, and adolescence is considered a vulnerable period for experiencing SHA (Mountjoy et al., 2016). Lack of reporting systems and codes of practice in school and sports may communicate lack of protection and low priority of the adolescents’ safety, possibly increasing the risk of SHA victimization and decreasing disclosure (Mountjoy et al., 2016). Among American undergraduate students, it has been reported that four out of five were aware of their schools’ sexual harassment policies, and more than half of the students knew who to contact if they experienced SHA (Hill and Silva, 2005). In the sport context, one out of five American student-athletes expressed knowledge about where to report SHA (Adhia et al., 2023), and up to 70% of athletes who responded to a questionnaire during the Youth Olympic Games were aware of where they could report or get help if they experienced or witnessed situations with harassment or abuse (Mountjoy et al., 2020, 2021b). Similar research among coaches and administrators is limited. One study addressed sexual harassment issues among American physical education staff and reported that 67% of athletic trainers were aware of their institution’s sexual harassment policy, but less than half understood the content (Velasquez, 1998).

There is a complex interaction between personal- and contextual factors that affect a person’s choice to disclose experiences of SHA or not. Barriers comprise fear of consequences; not being believed; losing social support; shame and guilt; and limited knowledge about where and how to report (Hill and Silva, 2005; Mountjoy et al., 2016; Lemaigre et al., 2017; Morrison et al., 2018; Hafstad and Augusti, 2019; Solstad, 2019). Therefore, it is not uncommon that people hide their experiences of SHA for many years, or do not disclose at all (Steine et al., 2016). Between 18–56% of middle- and high school students (Priebe and Svedin, 2008; Hill and Kearl, 2011; Hafstad and Augusti, 2019), college students (Hill and Silva, 2005), and adult athletes (Kerr et al., 2019; Hartill et al., 2021) do not disclose SHA. Of those who do, most of them are girls reporting to friends/peers and family members (Hill and Silva, 2005; Priebe and Svedin, 2008; Hill and Kearl, 2011; Hartill et al., 2021).

Disclosure of SHA is an important pathway to reduce the sequalae of SHA incidents, and here the presence of school-based report- and support resources is essential. However, awareness about the existence and use of such resources in the sport school context is not known, neither among adolescent athletes nor among leaders and coaches. Hence, this study is pioneering in Norway. Addressing SHA in school requires proactive leaderships and policy interventions (Hartill et al., 2021). Yet, there is a common thought that increasing awareness about a theme will have a preventive effect *per se*, but this “natural effect” of the schools’ participation in a study about SHA and available resources remains to be tested.

Therefore, this study aimed to examine:

1. awareness and use of reporting systems and support mechanisms for SHA in the school system among adolescent elite athletes, recreational athletes, and reference students at baseline (T1) and follow-up (T2), and whether there was a difference in awareness of these resources between adolescents with and without experience of SHA.
2. awareness of reporting systems and support mechanisms for SHA in the school system among leaders and coaches at T1.
3. the proportion of adolescents who disclosed their experiences with SHA at T1 and T2, and who they disclosed to in the school setting.
4. if the adolescents perceived that measures for SHA were implemented or newly informed in the participating schools at T2.

## Methods

### Design, recruitment, and procedure

This study was a part of a large prospective study of the prevalence of SHA, conducted in October 2019–May 2020 (T1) and October 2020–March 2021 (T2) (Sølvberg et al., 2022). All Norwegian sport high schools and a sample of non-sport high schools were invited to participate in the study by e-mail and follow-up telephone calls. School visits were arranged with the participating schools, where the adolescents responded to an online questionnaire during a school lesson. The leaders and coaches responded to a different version of the questionnaire.

### Participants

#### Adolescents

**Figure 1:**
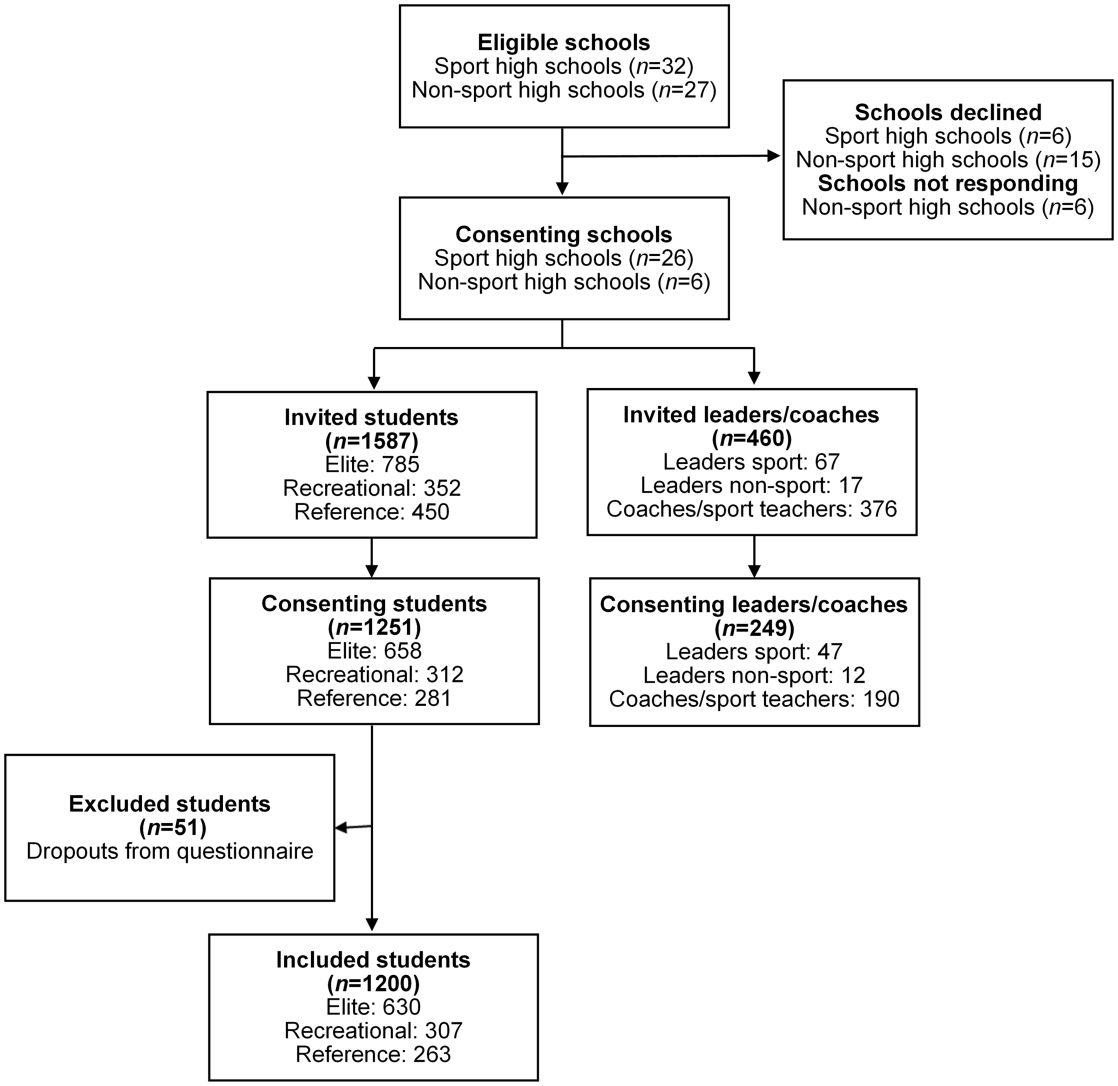
Flow chart of the recruiting process of adolescents, leaders, and coaches. All consenting schools were represented by the included students and coaches/leaders.

The inclusion and exclusion of schools and participants are described in Figure 1. Adolescents in 12th grade (mean age: 17.1 years, SD: 0.4) at the participating sport- and non-sport high schools were invited. The response rate was 78.8%. After excluding dropouts, the final sample of 1,200 adolescents were grouped into three groups (elite athletes, recreational athletes, and reference students) depending on which school they attended. The elite athletes attended private elite sport high schools or public sport high schools with specified elite sport programs. These schools collaborate with the Norwegian Olympic Sport Center, providing opportunities to combine education with high-level sport development and performance [Kristiansen and Houlihan, 2017; Olympiatoppen (The Norwegian Olympic and Paralympic Committee and Confederation of Sports), 2019; Thompson et al., 2022]. Public sport high schools in Norway also offer general sport education programs. Adolescents attending these programs were classified as recreational athletes in this study. The reference students attended educational programs without sport specialization and were recruited from other public high schools than the athletes, defined as non-sport high schools in this study.

#### Leaders and coaches

We invited leaders (i.e., principals, administrators, and heads of sports) and coaches with minimum 20% employment status at the sport high schools (Figure 1). Some of the leaders had dual roles as coaches and were counted as the role they self-reported in the questionnaire. The total response rate was 54.1%.

### Questionnaire

#### Sexual harassment and abuse

SHA was defined in the questionnaire as “(…) any form of unwanted sexual attention that has the purpose or effect of being offensive, frightening, hostile, degrading, humiliating or troublesome” (The Norwegian Equality and Anti-Discrimination Act § 13). Thirteen questions were listed covering a continuum of verbal (e.g., unwanted sexual comments/remarks), non-verbal (e.g., having sexual pictures/videos of oneself shared without consent), and physical sexual experiences (e.g., unwanted physical contact, rape). The participants were asked to respond to whether they had experienced these situations themselves or not (yes/no). Details are described elsewhere (Sølvberg et al., 2022). Those who reported yes to minimum one of the items, regardless of social setting, were classified as having experienced SHA. At T1, the questions covered lifetime experiences, and at T2 the questions covered 12-months experiences of SHA.

#### Disclosure

Disclosure of SHA was measured with a single item, i.e., “Did you disclose to someone about your experiences?” (yes/no). If yes, a follow-up question asked; “who did you disclose to?” with the options friends/peers, coach/teacher, employer, health personnel, parents/family, others, and reporting system. Multiple responses were possible.

#### Reporting system and support mechanisms

Awareness of the schools’ reporting system and support mechanisms was measured by the following two items: “Does your school have procedures/systems for reporting sexual harassment?” (yes/no/I do not know) and “Does your school have emergency procedures and/or support mechanisms for people who experience sexual harassment or report/disclose experiences with sexual harassment?” (yes/no/I do not know). A follow-up question was given to those who indicated that their school had a support system: “Would you use this support system?”. Negative responses were followed with an optional comment box to describe reasons for not wanting to use the system. The comments were categorized and quantified by counting the number of comments in each category.

#### Implementation of SHA measures in school

The adolescents were asked at T2 if their school had implemented measures related to SHA/unwanted sexual experiences the last year. If “yes,” a voluntary comment box to describe these measures was provided and the same quantifying method as described above was applied.

#### Leaders and coaches

The leaders and coaches answered a questionnaire including demographic questions, educational background, sports- and coaching experience, and the same questions about report- and support resources as the adolescents.

The student version and the adult version of the questionnaire were created in SurveyXact (Ramböll, Aarhus, Denmark). The student version was piloted by six adolescents and the adult version was piloted by three leaders/coaches at high schools not relevant for inclusion in the final study. Following the pilots, small wording adjustments were done to specify interpretation of the questions. After the first data collection, each school received a report with results of experiences with SHA and awareness about report- and support resources at their school.

### Statistical analyses

SPSS version 28.0 (IBM, Armonk, New York, United States) was used for statistical analyses. No power calculation was performed because we invited the total population of elite athletes at Norwegian sport high schools. ANOVA with Bonferroni post-hoc test was used to analyze differences in numerical data for boys, and Welch test with Games-Howell post-hoc test for girls due to unequal variances. Effect sizes were presented as Eta-squared for numerical data. Categorical data were analyzed using Pearson Chi-Square test for independence with Phi or Cramer’s V effect sizes. McNemar test was used to analyze changes from T1 to T2. Value of *p*≤0.05 were considered statistically significant.

The adolescents reporting non-binary gender identification were excluded from the analyses regarding gender differences because of a low number of participants (*n* = 4). For the questions about reporting systems and support mechanisms, the response options “no” and “I do not know” were merged for analytic purpose in the prospective analyses. For analyses related to aim 1 and 2, we used the total sample of adolescents and leaders/coaches, respectively. The analyses for aim 3 were based on the subsample of adolescents having experienced SHA (*n* = 696) as only those who reported experience of SHA received follow-up questions about disclosure. The prospective analyses were conducted with the subsample of adolescents who responded to both T1 and T2 on disclosure (*n* = 308), report- and support resources (*n* = 907), and preventive measures (*n* = 907).

### Ethics statement

The study was approved by the Regional Committees for Medical and Health Research Ethics (REC) (No. 8673), The Norwegian Center for Research Data (NSD) (No. 960987), registered in Clinical Trials (NCT04003675), and conducted according to the Helsinki Declaration. The participants were informed about the possibility to withdraw at any time without personal consequences. Electronic informed consent was signed by all participants. Adolescents above 16 years in Norway can consent to participate in health-related research without parental consent (Norwegian Health Research Act §17). Collected data were de-identified. Contact information to the project leader and the school nurse was distributed, encouraging the adolescents to talk to someone if they had concerns regarding the themes of this study. A specific question at the end of the questionnaire asked if the participant needed to talk to someone. Students who replied “yes” were contacted by the project leader.

## Results

### Sample characteristics

Characteristics of the adolescent sample are presented in Table 1. In total, 58.0% of the adolescents reported lifetime experience of SHA at T1 (50.5% elite athletes, 66.8% recreational athletes, and 65.8% reference students). The mean age of the leaders was 45.9 (SD = 6.7) years and 52.0 (SD = 8.1) years at non-sport and sport high schools, respectively, and one third were males. The coaches were on average 41.7 (SD = 10.3) years and 79.5% were males. Most coaches (79.5%) reported a sports-specific educational background and had been employed at a sport high school for an average of 8.4 (SD = 7.2) years. Two thirds were also coaching in their free time outside of school.

**Table 1:** Baseline (T1) characteristics of the adolescent sample.

	Elite athletes	Recreational athletes	Reference students	value of *p*	Effect size, *η*^2^/*V*
**Boys (*n* = 601, 50.1%)**	(*n* = 334)	(*n* = 178)	(*n* = 89)		
Heterosexual orientation, *n* (%)	327 (97.9)	178 (100.0)	84 (94.4)	n/a	n/a
Living with one or two parents, *n* (%)	211 (63.2)^a^	152 (85.4)^b^	81 (91.0)^b^	≤0.001	*V*: 0.275
First- or second-generation immigrants, *n* (%)	13 (3.9)^a^	21 (11.8)^b^	18 (20.2)^b^	≤0.001	*V*: 0.211
Training sessions per week, mean (SD)	10.6 (3.1)^a^	9.5 (3.2)^b^	4.2 (2.9)^c^	≤0.001	*η*^2^: 0.332
Training hours per week, mean (SD)	16.8 (5.5)^a^	15.7 (6.1)^a^	6.9 (5.7)^b^	≤0.001	*η*^2^: 0.266
Competitive sport, *n* (%)	324 (97.0)^a^	147 (82.6)^b^	18 (20.2)^c^	≤0.001	*V*: 0.675
**Girls (*n* = 595, 49.6%)**	(*n* = 294)	(*n* = 127)	(*n* = 174)		
Heterosexual orientation, *n* (%)	280 (95.2)	123 (96.9)	163 (93.7)	0.447	*V*: 0.052
Living with one or two parents, *n* (%)	195 (66.3)^a^	114 (89.7)^b^	162 (93.1)^b^	≤0.001	*V*: 0.314
First- or second-generation immigrants, *n* (%)	16 (5.4)^a^	4 (3.1)^a^	30 (17.2)^b^	≤0.001	*V*: 0.207
Training sessions per week, mean (SD)	9.6 (2.4)^a^	8.8 (3.5)^b^	5.1 (3.5)^c^	≤0.001	*η*^2^: 0.295
Training hours per week, mean (SD)	15.6 (5.7)^a^	14.2 (5.4)^b^	7.0 (5.4)^c^	≤0.001	η^2^: 0.315
Competitive sport, *n* (%)	285 (96.9)^a^	97 (76.4)^b^	45 (25.9)^c^	≤0.001	*V*: 0.679

*η*^2^, Eta-squared effect size; V, cramer’s V effect size; n/a, not applicable – statistical assumptions violated.

^a,b,c^: subscripts with different letters indicate significant differences between the school groups. *n* = 4 missing (non-binary gender).

### Reporting systems

In total, 11.4% of the adolescents reported that their school had a reporting system for SHA. A higher proportion of adolescent boys were aware of the reporting system compared to girls (14.0% vs. 8.9%, X^2^ (2, 1,196) = 8.243, *p* = 0.016, Phi = 0.083). There were no differences in awareness of reporting systems between the elite athletes, the recreational athletes, and the reference students (X^2^ (4, *n* = 1,200) = 7.651, *p* = 0.105, Cramer’s *V* = 0.056) (Table 2), or between the adolescents with experience of SHA and those without experience of SHA (11.2% vs. 11.7%, X^2^ (2, n = 1,200) = 0.072, *p* = 0.788, Phi = 0.008).

**Table 2:** Adolescents’, leaders’, and coaches’ awareness of reporting systems and support mechanisms in their school system.

	Adolescents			Leaders		Coaches*
	Elite athletes (*n* = 630)	Recreational athletes (n = 307)	Reference students (*n* = 263)	Sport high schools (*n* = 47)	Non-sport high schools (*n* = 12)	Sport high schools (*n* = 189)
Reporting systems						
Yes	72 (11.4)	45 (14.7)	20 (7.6)	44 (93.6)	12 (100.0)	98 (51.9)
No	106 (16.8)	51 (16.6)	41 (15.6)	2 (4.3)	–	6 (3.2)
Do not know	452 (71.7)	211 (68.7)	202 (76.8)	1 (2.1)	–	85 (45.0)
Support mechanisms						
Yes	199 (31.6)	123 (40.1)	86 (32.7)	45 (95.7)	11 (91.7)	97 (51.3)
No	40 (6.3)	19 (6.2)	22 (8.4)	-	1 (8.3)	1 (0.5)
Do not know	391 (62.1)	165 (53.7)	155 (58.9)	2 (4.3)	-	91 (48.1)

The results are presented from baseline data (T1) as number (percentages). *n = 1 missing coach.

### Support mechanisms

A total of 34.0% of the adolescents reported that their school had a system offering help and support to those experiencing or reporting SHA. There were no differences in awareness of support mechanisms between boys and girls [X^2^ (2, *n* = 1,196) = 0.035, *p* = 0.982, Phi = 0.005], between the three school groups [*X*^2^ (4, *n* = 1,200) = 8.256, *p* = 0.083, Cramer’s *V* = 0.059] (Table 2), or between adolescents with and without experience of SHA [34.8% vs. 32.9%, *X*^2^ (2, *n* = 1,200) = 0.438, *p* = 0.508, Phi = −0.019].

**Figure 2:**
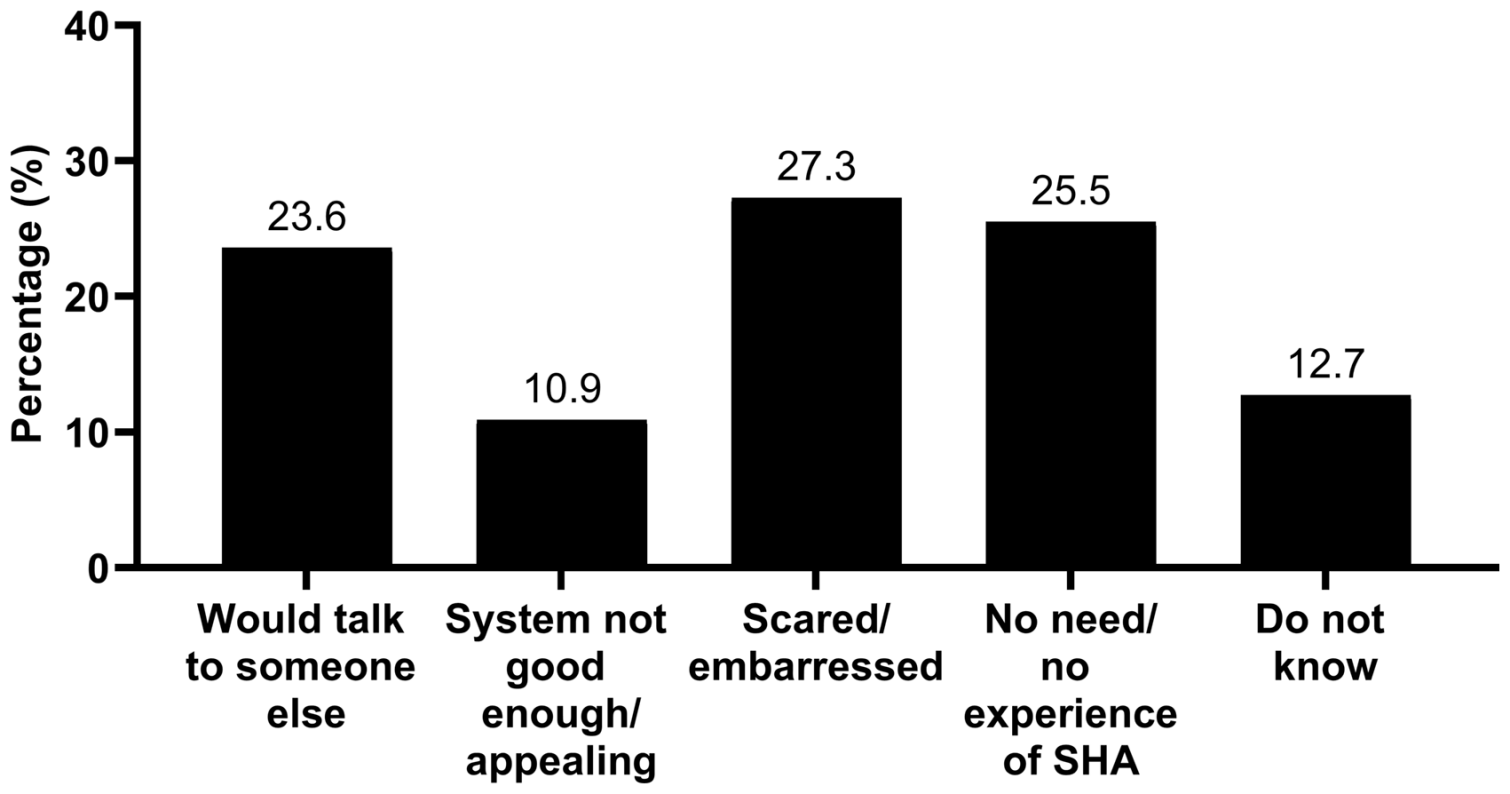
Reported reasons for not using the support system in school. *n* = 55 adolescents who choose to comment upon why they would not consider using the support system. SHA, sexual harassment and abuse.

Among the adolescents who were familiar with the support mechanisms, 53.2% reported that they would use them if they needed help, 11.5% would not, and 35.3% were not sure. There were no differences between boys and girls [*X*^2^ (2, *n* = 405) = 3.796, *p* = 0.150, Phi = 0.097] or between school groups [*X*^2^ (4, *n* = 408) = 1.819, *p* = 0.769, Cramer’s *V* = 0.047] regarding their consideration to use the support mechanisms. Reported reasons for not wanting to use the system are specified in Figure 2.

### Change in awareness of reporting systems and support mechanisms from T1 to T2

Among those who responded to the items regarding awareness of reporting systems at both time points (*n* = 907), a similar proportion of adolescents changed their response from “yes” at T1 to “no/I do not know” at T2 (6.4%), compared to those who changed from “no/I do not know” at T1 to “yes” at T2 (8.6%) (*p* = 0.103). A higher proportion of adolescents changed their response regarding awareness of support mechanisms from “yes” at T1 to “no/I do not know” at T2 (18.1%) compared to the opposite (13.9%) (*p* = 0.030).

### Leaders’ and coaches’ awareness of report- and support resources

All leaders, except three, responded that their school had report- and support resources for SHA. Among the coaches, 51.9 and 51.3% were aware of the reporting system and the support mechanisms, respectively (Table 2). Only descriptive data are provided for the leaders/coaches as statistical assumptions were not satisfied for further analyses.

### Disclosure

**Figure 3:**
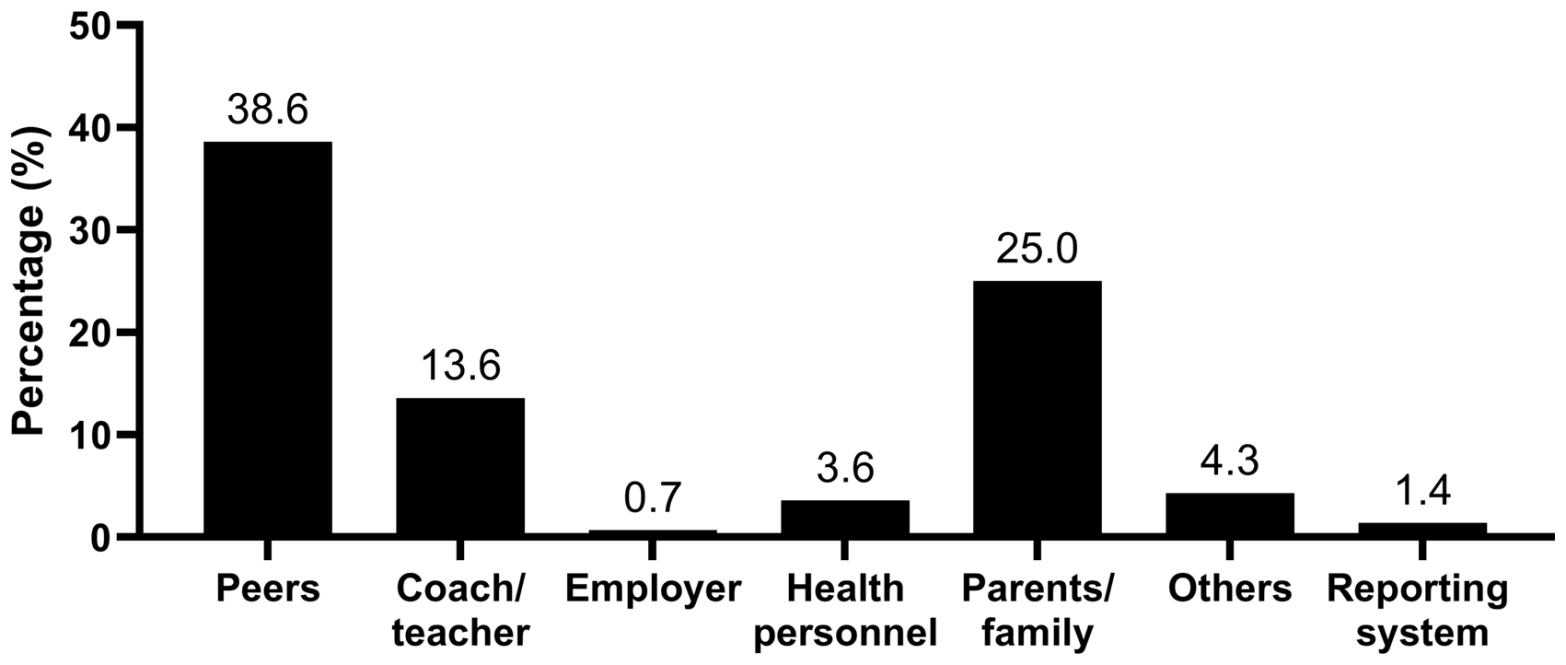
Reported points of disclosure of sexual harassment and abuse in the school setting (*n =* 140). The participants could mark several options. The numbers do not add up to 100% because the participants could report points of disclosure in other settings not reported here (sport setting and free time setting).

Among the adolescents who reported lifetime experiences of SHA (*n* = 696, 58%), regardless of which social setting they experienced it, 20.1% had disclosed to someone. Girls disclosed more often than boys [25.4% vs. 12.1%, *X^2^* (2, *n* = 693) = 18.356, *p* ≤ 0.001, Phi = −0.163]. A lower percentage of the elite athletes (18.6%) and the recreational athletes (16.1%) disclosed compared with the reference students (27.7%) [*X^2^* (2, *n* = 696) = 8.810, *p* = 0.012, Cramer’s *V* = 0.113]. The adolescents who had experienced SHA in the school setting mainly disclosed to peers, parents/family, or teachers/coaches (Figure 3). Two adolescents reported having used the reporting system in their school.

### Change in disclosure from T1 to T2

Among the adolescents who reported experience of SHA at both T1 and T2, and thereby responded to the question about disclosure (*n* = 308), 7.5% disclosed at both time points, while 67.5% had not disclosed at all. A higher proportion of adolescents disclosed at T1 but not on T2 (17.9%) compared to those who did not disclose at T1 but disclosed at T2 (7.1%) (*p* ≤ 0.001).

### Implemented measures against sexual harassment and abuse in school between T1 and T2

Among the adolescents who responded to the questionnaire at T2 (*n* = 907), 59 (6.5%) reported that their school had implemented measures or informed about unwanted sexual attention the last year. There was no difference between boys and girls [X^2^ (2, *n* = 903) = 1.118, *p* = 0.290, Phi = 0.035] or between the school groups [X^2^ (2, *n* = 907) = 3.057, *p* = 0.217, Cramer’s *V* = 0.058]. The measures reported by the adolescents were presentations/information about SHA (33.9%), information about support/help (30.5%), surveys (23.7%), and individual-tailored measures (5.1%). Lastly, 6.8% did not provide details.

## Discussion

We examined high school students’ disclosure of SHA, and awareness of report- and support resources in school among students, leaders, and coaches. Overall, few adolescents disclosed experiences of SHA, with elite- and recreational athletes disclosing less than reference students. The awareness of available resources was limited among adolescents and coaches.

### Report- and support resources

Compared with previous studies (Hayes-Smith and Levett, 2010; Walsh et al., 2010; Adhia et al., 2023) we found a lower proportion of adolescents being aware of reporting systems for SHA in school, yet a higher proportion being aware of support mechanisms (Hayes-Smith and Levett, 2010). Unfamiliarity with such resources may be a result of lack of information provided from the schools or limited attention from the adolescents when the information was provided. Experience of SHA did not seem to motivate adolescents in this study to seek information about report- and support resources as no differences were found between adolescent with and without experience of SHA. Nevertheless, information alone may not be enough if the resources are not being used (Hayes-Smith and Levett, 2010). Our finding that the adolescents do not consider the support system good enough, and only half of them would use the system if they needed help, may reveal that the adolescents do not see the school health service a place to go for support related to SHA victimization.

The result that a higher proportion of adolescent boys compared to girls were aware of the school’s reporting system was significant, but small differences in actual percentages and a small effect size emphasize low relevance of this difference.

Regarding the negative change in awareness of support mechanisms, it is unlikely that the students who reported knowing the system at T1 had forgotten about it 1 year later. Rather, in the T1-T2 interval, the COVID-19 pandemic forced schools to lock down and the transference of school nurses to other health care services may have reduced the adolescents’ access to health care services in school (Granrud and Leonhardt, 2021).

### Leaders’ and coaches’ awareness of report- and support resources

There is a lack of research covering awareness of SHA policies and resources among coaches and school leaders. As it is the responsibility of adults to facilitate a safe school- and sport environment for adolescents (Tuakli-Wosornu et al., 2023), it was worrying that only half of the coaches in this study were aware of their schools’ report- and support system. Our finding that school leaders were more aware of SHA resources in school than the coaches aligns with previous studies reporting that policies are less known, or unknown, at lower levels in the sport system (Parent, 2011).

### Disclosure

The percentage of adolescents in this study who disclosed experiences with SHA was lower than reported elsewhere among non-sport adolescents (Hill and Silva, 2005; Priebe and Svedin, 2008; Hill and Kearl, 2011) and adult athletes (Kerr et al., 2019; Hartill et al., 2021; Mountjoy et al., 2021a), yet higher than student-athletes (Adhia et al., 2023). The latter study examined only formal reports filed to the institution, likely resulting in lower rates of disclosure. Low rates of formal disclosure have also been reported in previous research among adolescents in general (Priebe and Svedin, 2008; Hafstad and Augusti, 2019) and among athletes (Kerr et al., 2019; Hartill et al., 2021; Mountjoy et al., 2021a). The other studies involving athletes have older samples (Kerr et al., 2019; Mountjoy et al., 2021a) or have retrospectively surveyed adults about their experiences with SHA as children (Hartill et al., 2021). Compared to the adolescent sample in this study, and the fact that victims of SHA tend to delay disclosure (Hébert et al., 2009; Steine et al., 2016), it is likely that the rate of disclosure is higher in studies with adult samples as they have lived longer and have had more time to process the incidence.

The reported points of disclosure align with findings in other studies (Priebe and Svedin, 2008; Walsh et al., 2010; Hartill et al., 2021; Mountjoy et al., 2021a). Increased significance of friendships in adolescence might be reflected in the result that peers was the most frequent recipient of disclosures. Adolescents may feel more comfortable talking to same-aged peers than talking to adults (Priebe and Svedin, 2008), also underlined by the reasons provided in this study for not wanting to use the support system in school like feeling “more comfortable talking to someone else.”

The fact that elite- and recreational athletes disclosed less often than the reference students support former research highlighting sport-specific barriers to disclosure like fear of consequences for their sport career, losing their “tough” sport identity, or losing social belonging (Mountjoy et al., 2016; Bjørnseth and Szabo, 2018; Kerr et al., 2019; Solstad, 2019). Athletes may be socialized into a sports environment where inappropriate behaviors and comments become normalized. Consequently, situations may not be disclosed although it might have negative personal impacts (Toftegaard, 2001; Parent and Fortier, 2018).

We cannot explain the observed gender difference in disclosure from the results in this study, but it corresponds to other researchers’ findings (Priebe and Svedin, 2008; Hébert et al., 2009; Walsh et al., 2010; Mountjoy et al., 2021a), and could be related to underreporting of SHA among boys and the stereotype that girls are more prone to seek help for health-related issues (Lemaigre et al., 2017). The societal norm of male masculinity and toughness in sport may be another barrier to disclose SHA for male athletes as some forms of harassment might be considered harmless and natural for boys (Hartill et al., 2021), and showing vulnerability and emotions may be judged as a weakness (Sivagurunathan et al., 2019). Male victims of SHA are also suggested to blame themselves for not hindering the incidence, possibly preventing them from disclosing (Hébert et al., 2009). Striving to fit into a peer group may reinforce barriers and maintain a culture of silence, especially in adolescents.

### Implemented measures against sexual harassment and abuse

Considering the results in this study, reflecting the content in the reports received by the schools, it is unfortunate that few adolescents reported at T2 that their school had implemented measures against SHA. However, it corresponds with our findings regarding no change in awareness of reporting procedures, and even a negative change in awareness of support systems. If measures had been implemented in school, it would have been likely that the adolescents’ awareness of resources also would increase (Hayes-Smith and Levett, 2010). The schools spent much time restructuring the school day and adapt to online teaching during COVID-19 lockdown. Nevertheless, we believe that information about SHA and available resources could have been provided digitally in all schools.

### Strengths and limitations

The main strengths are the inclusion of a large and representative sample of adolescent athletes and non-athletes from different geographical parts of Norway, as well as the inclusion of coaches and leaders, and a high response rate. Limitations are connected to wordings and structure of the questionnaire. If the adolescents linked the word “disclosed” to formal disclosure only and responded “no,” follow-up questions about less formal points of disclosure would be left out. Additionally, methodological factors may also account for the negative change in disclosure as we examined lifetime SHA and disclosure at T1 and 12 months SHA and disclosure at T2. Willingness to disclose could be affected by factors which was not included in this paper like type of SHA, frequency, and relation to the perpetrator.

### Implications and future studies

Actions should be taken to increase awareness about relevant report- and support resources to close the gap from victimization to disclosure and help-seeking. Such resources should be accessible and adapted to resonate with adolescents’ needs, highlighting the importance of user involvement. Peers may be the first recipient of information about SHA victimization, indicating a need to include peer support in preventive programs for SHA. Concerning our findings of different rates of disclosure between athletes and reference students, future studies should closely examine sport-specific barriers to disclosure.

## Conclusion

Awareness of available report and support resources in school was limited among adolescents and coaches, but satisfactory among school leaders. One in five adolescents disclosed experiences of SHA, mainly to peers and family members. Most of the adolescents did not notice if their school implemented any measures to reduce SHA or increase awareness about available resources. These results call for increased institutional effort, ideally with adolescent’ user involvement, to lower barriers for help-seeking and relevance of available resources.

## Data availability statement

The raw data supporting the conclusions of this article will be made available by the authors, without undue reservation.

## Ethics statement

The studies involving human participants were reviewed and approved by the Regional Committees for Medical and Health Research Ethics (REC) (No. 8673). Written informed consent from the participants’ legal guardian/next of kin was not required to participate in this study in accordance with the national legislation and the institutional requirements.

## Author contributions

NS and JS-B collected the data. NS wrote the first draft of the manuscript. All authors contributed to the planning of the project, initiated by JS-B, revision of the manuscript and approved the final version.

## Funding

The project was funded by the Foundation Dam via The Norwegian Women’s Public Health Association. The funding sources were not involved in designing the study; the data collection; analyses and interpretation of the data; writing the paper; or the submission process. Open access publishing was supported by the Department of Sports Medicine at the Norwegian School of Sport Sciences.

## Conflict of interest

The authors declare that the research was conducted in the absence of any commercial or financial relationships that could be construed as a potential conflict of interest.

## Publisher’s note

All claims expressed in this article are solely those of the authors and do not necessarily represent those of their affiliated organizations, or those of the publisher, the editors and the reviewers. Any product that may be evaluated in this article, or claim that may be made by its manufacturer, is not guaranteed or endorsed by the publisher.

## Abbreviations

SHA: sexual harassment and abuse
